# A novel PKD2L1 C-terminal domain critical for trimerization and channel function

**DOI:** 10.1038/srep09460

**Published:** 2015-03-30

**Authors:** Wang Zheng, Shaimaa Hussein, JungWoo Yang, Jun Huang, Fan Zhang, Samuel Hernandez-Anzaldo, Carlos Fernandez-Patron, Ying Cao, Hongbo Zeng, Jingfeng Tang, Xing-Zhen Chen

**Affiliations:** 1 Membrane Protein Disease Research Group, Department of Physiology, Faculty of Medicine and Dentistry, University of Alberta, T6G 2H7, Edmonton, AB, Canada; 2 Department of Chemical and Materials Engineering, Faculty of Engineering, University of Alberta, T6G 2V4, Edmonton, AB, Canada; 3 School of Life Sciences and Technology, Tongji University, 200092, Shanghai, China; 4 Department of Biochemistry, Faculty of Medicine and Dentistry, University of Alberta, T6G 2H7, Edmonton, AB, Canada; 5 Membrane Protein Disease and Cancer Research Center, Hubei University of Technology, 430068, Wuhan, Hubei, China

## Abstract

As a transient receptor potential (TRP) superfamily member, polycystic kidney disease 2-like-1 (PKD2L1) is also called TRPP3 and has similar membrane topology as voltage-gated cation channels. PKD2L1 is involved in hedgehog signaling, intestinal development, and sour tasting. PKD2L1 and PKD1L3 form heterotetramers with 3:1 stoichiometry. C-terminal coiled-coil-2 (CC2) domain (G699-W743) of PKD2L1 was reported to be important for its trimerization but independent studies showed that CC2 does not affect PKD2L1 channel function. It thus remains unclear how PKD2L1 proteins oligomerize into a functional channel. By SDS-PAGE, blue native PAGE and mutagenesis we here identified a novel C-terminal domain called C1 (K575-T622) involved in stronger homotrimerization than the non-overlapping CC2, and found that the PKD2L1 N-terminus is critical for dimerization. By electrophysiology and *Xenopus* oocyte expression, we found that C1, but not CC2, is critical for PKD2L1 channel function. Our co-immunoprecipitation and dynamic light scattering experiments further supported involvement of C1 in trimerization. Further, C1 acted as a blocking peptide that inhibits PKD2L1 trimerization as well as PKD2L1 and PKD2L1/PKD1L3 channel function. Thus, our study identified C1 as the first PKD2L1 domain essential for both PKD2L1 trimerization and channel function, and suggest that PKD2L1 and PKD2L1/PKD1L3 channels share the PKD2L1 trimerization process.

Eight members in the polycystic kidney disease (PKD) family of proteins have so far been identified: PKD1,-1L1, -1L2, -1L3, -REJ; PKD2, -2L1 and -2L2. Among them, only PKD1 and PKD2 are mutated in autosomal dominant PKD (ADPKD)^1^,^2^. PKD2, -2L1 and -2L2 form the transient receptor potential (TRP) polycystin (TRPP) subfamily and are called TRPP2, -3 and -5, respectively. Members of the TRP superfamily are cation channels and play critical roles in sensory physiology^3^,^4^. PKD2L1 and PKD2 share 50% sequence identity and 71% similarity and are predicted to have a similar membrane architecture: six transmembrane (TM) segments flanked by the intracellular amino- (N-) terminus (NT, M1-Y96) and carboxyl- (C-) terminus (CT, E566-S805), with a pore loop between TM5 and TM6. Sharing these topological features with other TRP members TRPP channels are presumably organized as homotetramers^5^,^6^,^7^. When over-expressed in *Xenopus* oocytes alone, PKD2L1 channel was found to traffic to the plasma membrane and be activated by Ca^2+^ and permeable to Ca^2+^^8^,^9^. PKD2L1 also acts as a Ca^2+^ channel on the mammalian cell surface membrane and primary cilia where it regulates Ca^2+^ concentration and Ca^2+^-dependent hedgehog signaling^10^,^11^. The same studies found that PKD2L1 knockout (KO) mice exhibit defective intestinal development. When over-expressed in human embryonic kidney (HEK) cells PKD2L1 is regulated by extracellular pH and cell swelling^12^. In addition, PKD2L1 is expressed in bipolar neurons in the tongue taste buds and neurons surrounding the central canal of spinal cord where it responds to a decrease in extracellular pH^13^. In fact, the same study found that genetic ablation of cells expressing PKD2L1 eliminates gustatory nerve response to sour stimuli in mice. The involvement of PKD2L1 in sour tasting was later confirmed using KO mouse models^14^.

There are theoretically 15 possible ways of complexing between a PKD1 and PKD2 homologue, which may nicely respond to various tissue-specific needs of sensory functions and regulations. Eg, the PKD2/PKD1 and PKD2L1/PKD1L3 channel complexes were reported to sense fluid flow and acid, in primary cilia and surface membrane, respectively^15^,^16^. PKD1 and PKD2L1 were also reported to form a channel complex when co-expressed in HEK cells, but the physiological role has remained unknown^17^. At least when co-expressed in HEK cells, PKD2L1 also interacts with PKD1L3 such that both efficiently traffic to the surface membrane where they act as an extracellular acid-induced off-response cation channel, ie, activation occurred only after low extracellular pH was removed^15^,^18^. Further, they are co-expressed in mouse circumvallate and foliate papillae although only PKD2L1 is found in other taste bud areas, including fungiform and palate taste buds^15^,^18^. These *in vitro* and *in vivo* data suggest that PKD2L1 and PKD1L3 are synergistically involved in acid sensing pathways. However, unlike PKD2L1 KO mice, PKD1L3 KO mice were not found to have defects in sour tasting^19^, arguing against the assumption that they are in the same pathway in the tongue. In primary cilia PKD2L1 and PKD1L1 form a Ca^2+^ channel that regulates the ciliary Ca^2+^ concentration and Ca^2+^-dependent hedgehog signaling pathway, which seems to be developmentally important^10^,^11^. In these studies, PKD1L1 was found to regulate the single-channel conductance of PKD2L1. However, it remains to be determined whether PKD1L1 KO mice also display similar developmental defects as those observed in PKD2L1 KO mice. Optimistically, more diverse functions and regulations associated with pairs of PKD proteins will be discovered in near future.

How does a PKD2 homologue complex with a PKD1 homologue? Studies using mammalian cell and *Xenopus* oocyte expression showed that both the PKD2/PKD1 and PKD2L1/PKD1L3 complexes form heterotetramers with 3:1 stoichiometry, ie, one PKD2 (or PKD2L1) trimer paired with one PKD1 (or PKD1L3) monomer^20^,^21^. Although no corresponding information for PKD2L1/PKD1 and PKD2L1/PKD1L1 is available it is reasonable to speculate that they are also organized with the same stoichiometry. The PKD2L1 C-terminal coiled-coil 2 domain (CC2, G699-W743) was found to homotrimerize *in vitro* and be important for PKD2L1 homomeric assembly and PKD2L1/PKD1L3 surface expression in *Xenopus* oocytes^21^,^22^,^23^. However, a previous study found that PKD2L1 truncation mutants lacking CC2 possess similar Ca^2+^-activated channel function as wild-type (WT) PKD2L1 and that PKD2L1 and PKD1L3 interact with each other through their TMs, which is required for PKD2L1/PKD1L3 surface expression in HEK cells^24^. This study also showed that cells still respond to 25 mM citric acid solution when co-expressed full-length PKD1L3 with PKD2L1 truncation mutant lacking either CC2 (PKD2L1ΔCC, M1-E653) or both CC2 and the EF-hand domain (PKD2L1ΔEF-CC, M1-F621), but little response was observed with PKD2L1 truncation mutant lacking the entire C-terminus (PKD2L1ΔCT, M1-I560), despite robust cell surface expression. This suggests the importance of a domain within I560-F621 for channel function^25^.

Based on the tetrameric assembly of PKD2^26^,^27^ and other TRP channels^5^,^6^,^7^ it is likely that PKD2L1 also possesses tetrameric organization. It may be speculated that a TRP homotetrameric channel is formed through dimerization of two homodimers. If such speculation would be true for PKD2L1, then it should possess two distinct domains, for dimerization of two monomers and of two homodimers, respectively, and these domains should also be distinct from a trimerization domain, eg, CC2. Unfortunately, it has so far remained unknown as to which PKD2L1 domains are critical for both its homomerization and channel function. In the present study, through the use of mutagenesis, non-reducing SDS-PAGE, blue native PAGE (BN-PAGE) and electrophysiology, among others, we discovered that a novel C-terminal domain called C1 (K575-T622) is essential for both homotrimerization and channel function, challenging the speculation that a functional homotetrameric TRP channel is formed through two consecutive steps of homodimerization.

## Results

### Oligomeric state of PKD2L1 in mouse tissues and human cells

**Figure 1:**
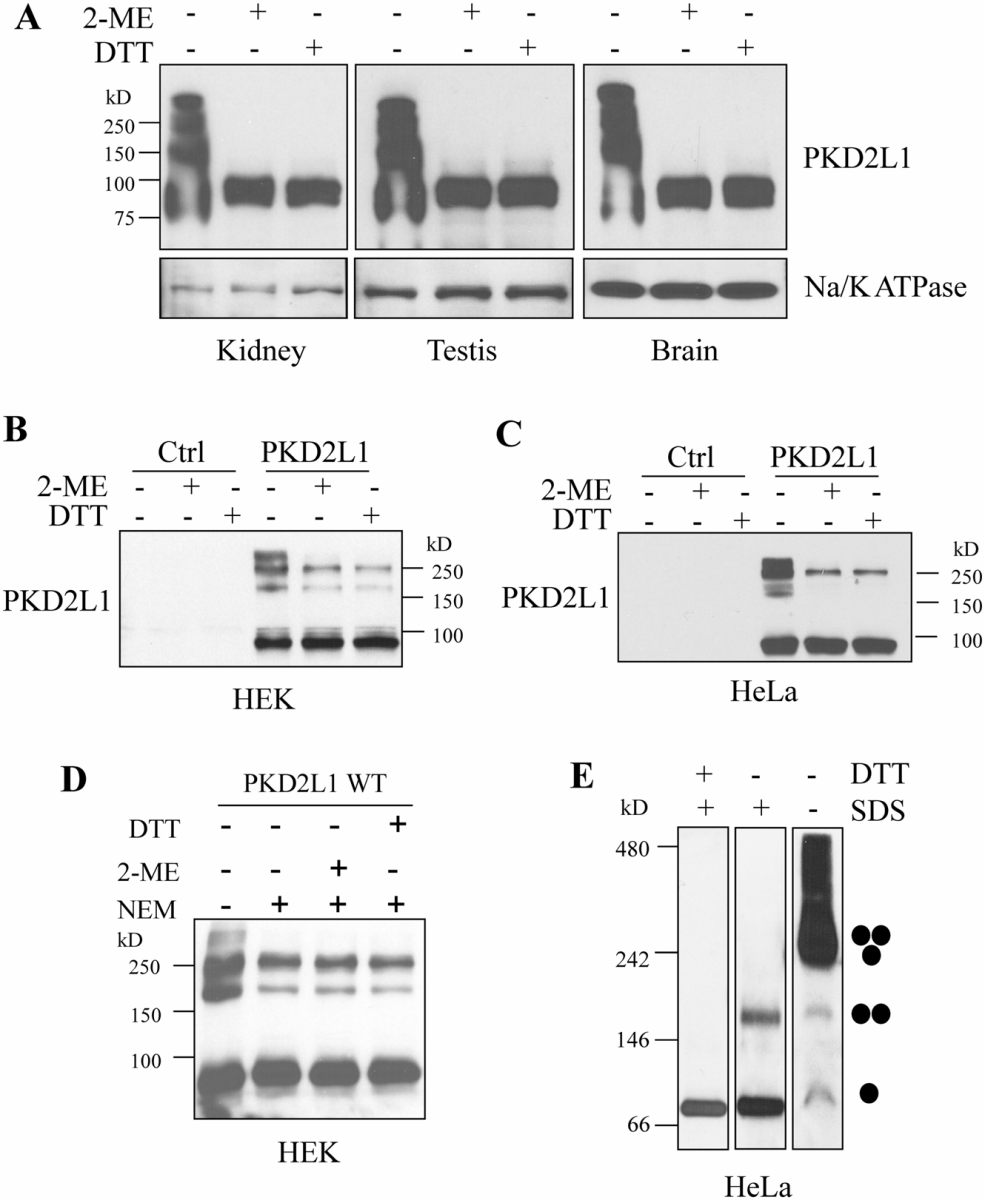
Oligomers of PKD2L1 in mouse tissues and human cell lines. (A) Detection of PKD2L1 in mouse kidney, testis, and brain tissues by WB. Samples were prepared with SDS loading buffer under the non-reducing and reducing (supplemented with 5% 2-ME or 0.5 M DTT) conditions. PKD2L1 was detected with an antibody from Abnova. Na/K ATPase was used as a loading control. Detection of over-expressed human PKD2L1 in HEK (B) and HeLa cells (C). pcDNA3.1(+) containing human PKD2L1 or empty vector (Ctrl) was transfected into HEK and HeLa cells. Cell lysates were collected 48 hr after transfection. Samples for SDS-PAGE were prepared under non-reducing or reducing condition. (D) Detection of over-expressed human PKD2L1 in HEK cells under non-reducing and reducing conditions. Samples were prepared with or without treatment by 10 mM NEM. (E) WB analysis after BN-PAGE of Flag-tagged PKD2L1 over-expressed in HeLa cells. Samples were prepared with or without 2.5% SDS, or with 2.5% SDS plus 0.1 M DTT. An anti-Flag antibody was used for WB detection. Putative PKD2L1 monomer, dimer and trimer are indicated.

Previous studies on PKD2L1 oligomerization were limited to *in vitro* conditions, including trimerization of its purified C-terminus or of the full-length protein over-expressed in *Xenopus* oocytes^21^,^22^,^23^. In order to determine the oligomeric states of PKD2L1 under more *in vivo* and physiological conditions, we performed Western Blot (WB) experiments using mouse tissues under the non-reducing and reducing conditions (see EXPERIMENTAL PROCEDURES). We found that in the kidney, testis and brain under the non-reducing condition, endogenous PKD2L1 displays similar oligomeric patterns from which the band sizes suggest the presence of either PKD2L1 homodimers, -trimers and –tetramers, or its heteromerization with endogenous interacting partner proteins (Fig. 1A). These oligomer bands disappeared in the presence of a reducing condition. To provide further documentations regarding whether these bands correspond to PKD2L1 homo- or heterooligomers we over-expressed human PKD2L1 in HEK (HEK293T) and HeLa cells in which partners of PKD2L1 were assumed to have little influence due to their absence or relatively low expression levels. In both HEK and HeLa cells under the non-reducing condition, we still detected four bands (Fig. 1B and C) that were comparable to the patterns obtained using mouse tissues. We thus tentatively assigned them as monomer, dimer, trimer and tetramer. Interestingly, under the reducing condition, a significant portion of trimers remained while all tetramers and the majority of dimers were absent. These data indicated that the oligomerization strength follows an order of trimer ≫ dimer > tetramer, consistent with a previous finding that the PKD2L1 CT forms very stable trimers *in vitro*^22^,^23^.

Because oligomerization may occur through a disulfide bond between two cysteine residues during lysate preparations or SDS-PAGE under denaturing conditions, and there are several cysteine residues in PKD2L1, we wondered whether disulfide bonds formed by these residues under oxidative conditions account for the observed oligomers. For this we included 10 mM N-ethylmaleinide (NEM) in cell lysis buffer to inhibit disulfide bond formation by modifying free cysteine residues. We found that the amount of oligomers decreases with the NEM treatment in HEK cells but does not further decrease with additional reducing agents (Fig. 1D), indicating that the observed oligomers in Fig. 1B and C are partially due to disulfide bond formation during sample preparations and/or electrophoresis, and that the oligomers detected in the presence of the NEM treatment are formed through peptide-peptide interactions.

We also employed BN-PAGE to check PKD2L1 oligomeric state under the non-denaturing condition. No disulfide bonds would be formed during sample preparation and/or electrophoresis with BN-PAGE. Our data indicated that PKD2L1 is mainly present as homotrimers, and possibly also as homotetramers or hetero-oligomers with its partner proteins, while monomers and dimers are still detectable under this condition (Fig. 1E). Only monomers were observed in the presence of SDS and dithiothreitol (DTT), while dimers were detectable if only SDS was applied (Fig. 1E). Previous studies showed that peptide-peptide interaction mediated oligomers of some membrane proteins, especially multiple-transmembrane proteins, can also be detected with non-reducing SDS-PAGE under denaturing conditions^28^,^29^,^30^ because SDS as a detergent may form micelles, similar to lipid vesicles, at certain concentrations to hold denatured oligomers by binding to hydrophobic transmembrane domains rather than to disrupting them into monomers^31^. However, since there is no SDS in the gel or running buffer on BN-PAGE, compared with SDS-PAGE, these oligomers/SDS micelle complexes may be dissociated once the samples are loaded into the gel. That may be the reason why no trimers were detected on BN-PAGE with SDS while trimers can still be observed on non-reducing SDS-PAGE. Taken together, our data using over-expressed PKD2L1 together with our *in vivo* data using mouse tissues strongly indicated that PKD2L1 forms homodimers, -trimers and -tetramers.

### Effects of intracellular N- and C-termini of PKD2L1 on its channel function and homomerization

**Figure 2:**
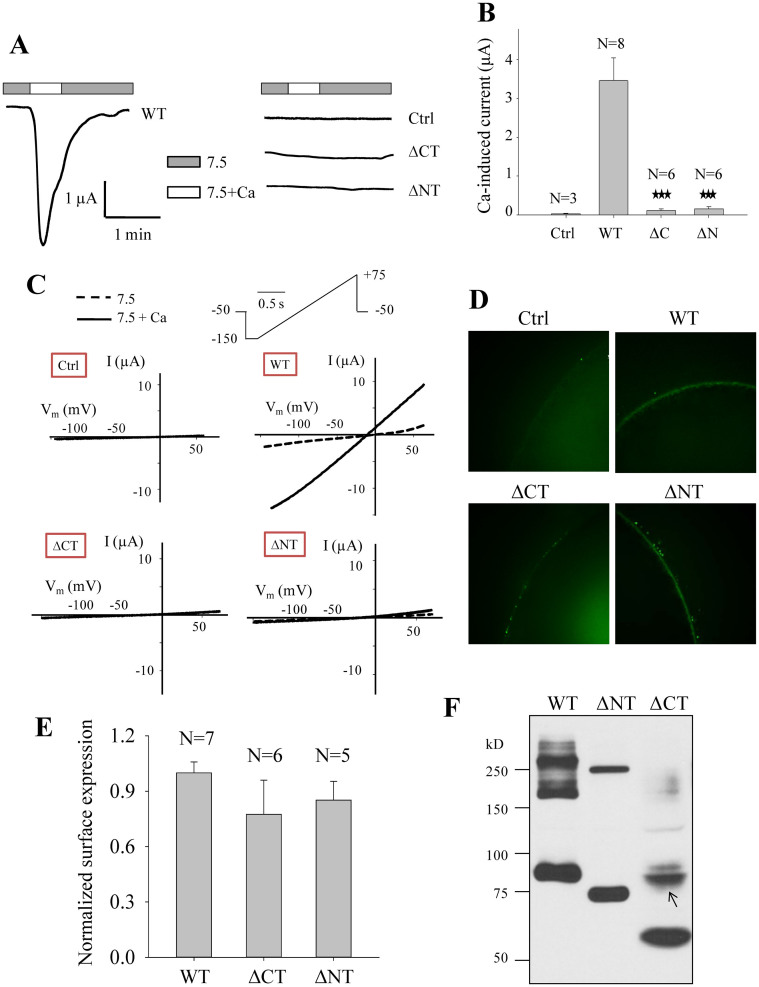
Roles of the human PKD2L1 N- and C-termini in its channel activity and oligomerization. (A) Representative whole-cell current tracings obtained from *Xenopus* oocytes expressing PKD2L1 WT, mutant ΔCT (deletion of E566-S805) or ΔNT (deletion of M1-Y96) using the two-microelectrode voltage clamp technique. Oocytes were voltage clamped at −50 mV. Data from a water-injected oocyte served as a negative control (Ctrl). Currents were measured using standard extracellular solution (pH 7.5) (7.5) or standard extracellular solution containing 5 mM CaCl_2_ (7.5+Ca). (B) Averaged currents obtained from oocytes expressing PKD2L1 WT, ΔCT, ΔNT or water (Ctrl). Currents were averaged from different numbers of oocytes, as indicated. ‘***' indicates p ≤ 0.001 when compared with the WT data. (C) Representative current–voltage relationship curves obtained using a voltage ramp protocol, as indicated, before (7.5) and after (7.5+Ca) addition of 5 mM CaCl_2_. (D) Representative immunofluorescence data showing the plasma membrane expression of PKD2L1 WT, ΔCT and ΔNT in oocytes. (E) Averaged and normalized surface expression of PKD2L1 WT, ΔCT and ΔNT in oocytes. Surface expressions were averaged from indicated numbers of oocytes and normalized to that of PKD2L1 WT. (F) WB detection of Flag-tagged human PKD2L1 WT, ΔNT and ΔCT over-expressed in HeLa cells under the non-reducing condition. A band (indicated by an arrow) that is unlikely a dimer based on its size remained unaccounted for was detected with the CT deletion.

PKD2L1 was first found to act as a Ca^2+^-activated non-selective cation channel when over-expressed in *Xenopus* oocytes in which PKD2L1 traffics to the plasma membrane^8^,^9^. Previous studies demonstrated that the PKD2L1 C-terminal domain CC2 formed a trimer *in vitro*^22^,^23^ but truncation mutant channels T622X and V670X that do not contain CC2 are still functional^24^, indicating that CC2 is not important for PKD2L1 channel function. On the other hand, mutant PKD2L1 lacking CT is still able to oligomerize^25^, indicating that a domain outside CT mediates the oligomerization. In an effort to identify domain(s) that are important for both PKD2L1 homomerization and channel function we first set to examine the effects of the PKD2L1 CT and NT on its channel function. For this we over-expressed PKD2L1 truncation mutant with deletion of CT (named ΔCT) or NT (named ΔNT) in *Xenopus* oocytes, and measured Ca^2+^-induced channel activation currents with the two-microelectrode voltage clamp. We found that both the ΔCT and ΔNT mutants are functionally dead although they continue to traffic to the plasma membrane (Fig. 2A–E), which is consistent with previous findings that PKD1L3/PKD2L1 ΔCT or PKD1L3/PKD2L1 ΔNT complex shows no response to 25 mM citric acid^25^.

**Figure 3:**
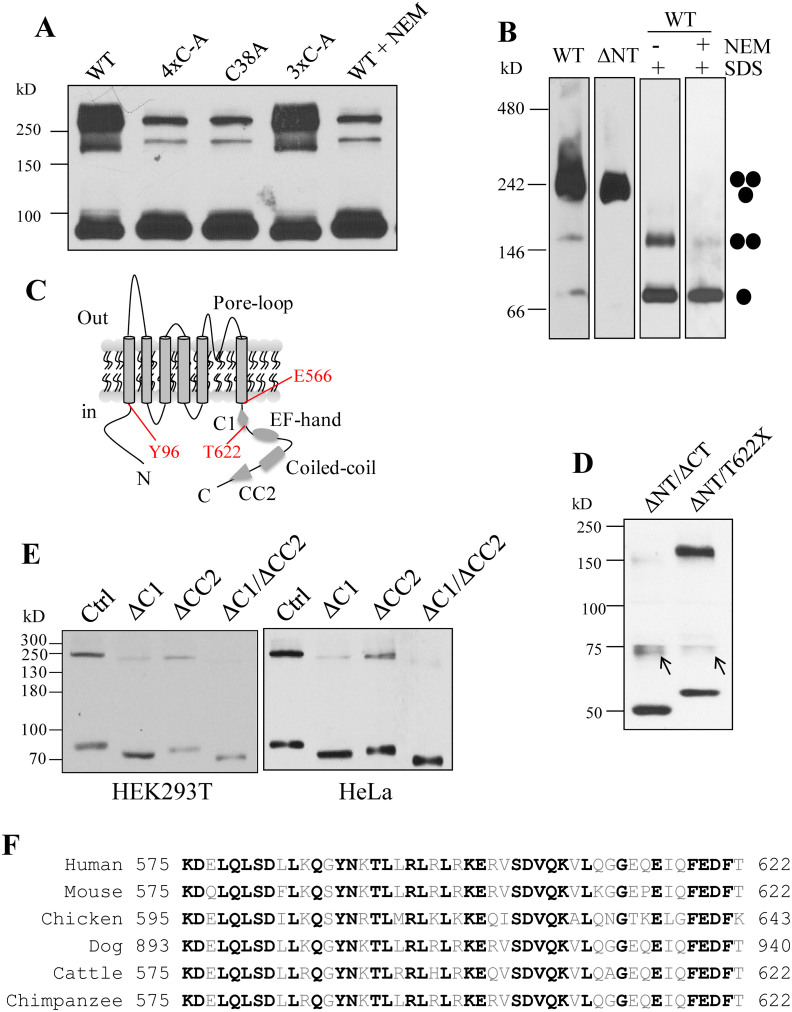
Identification of domain C1 of PKD2L1 critical for its trimerization. (A) WB detection of over-expressed Flag-tagged PKD2L1 WT, without or with 10 mM NEM treatment, or mutants 4×C-A (quadruple C38A, C60A, C70A and C74A mutations), C38A and 3×C-A (triple C60A, C70A and C74A mutations) under non-reducing condition. (B) WB analysis after BN-PAGE of Flag-tagged PKD2L1 WT and ΔNT over-expressed in HeLa cells. WT samples were prepared with or without 2.5% SDS, or with 2.5% SDS plus 10 mM NEM. Putative PKD2L1 monomer, dimer and trimer are indicated. (C) Schematic illustration of PKD2L1 membrane topology. TMs, pore-loop, C1, CC2, EF-hand, coiled-coil domains, and positions of residues Y96, E566 and T622 are indicated. (D) WB detection of Flag-tagged PKD2L1 mutants ΔNT/ΔCT (M1-Y96 and E566-S805 double deletion) and ΔNT/T622X (M1-Y96 and T622-S805 double deletion) over-expressed in HeLa cells under the non-reducing condition. Two unknown bands similar to one in Fig. 2F remained unaccounted for. (E) WB detection of over-expressed Flag-tagged PKD2L1 mutants in HEK or HeLa cells. All constructs were made from ΔNT. These included Ctrl (ΔNT), ΔC1 (C1, K575-T622, deletion from ΔNT), ΔCC2 (CC2, G699-W743, deletion from ΔNT), or ΔC1/ΔCC2 (C1 and CC2 double deletion from ΔN). (F) Amino acid sequence alignment of PKD2L1 C1 from indicated species. National Center for Biotechnology Information accession number for sequences used here are NP_057196 (human), NP_852087 (mouse), XP_426509 (chicken), XP_005637930 (dog), XP_002698535 (cattle), and XP_001168415 (chimpanzee). Identical residues among the species are indicated by black bold letters.

We next wanted to determine whether NT is important for oligomerization using HeLa cells. Absence of NT in mutant ΔNT abolished the oligomers except trimers with both SDS-PAGE (Fig. 2F) and BN-PAGE (Fig. 3B), suggesting that NT mediates homodimerization. Interestingly, deletion of CT resulted in loss of all trimers and most of dimers (Fig. 2F), suggesting the possibility that the dimerization is dependent, at least in part, on trimerization. Because we showed that part of dimers is due to disulfide bond formation during lysate preparations or SDS-PAGE under denaturing conditions (Fig. 1D), and there are four cysteine residues in NT, we wondered whether disulfide bonds formed by these residues under oxidative conditions account for the observed dimerization. For this we mutated one or more of these cysteine residues to alanine and found that mutation C38A, but not any of the other three mutations (C60A, C70A and C74A), substantially reduces the dimer band (Fig. 3A). Further, inclusion of NEM in cell lysis buffer exhibited a similar effect (Fig. 3A), which was confirmed with BN-PAGE (Fig. 3B). These data together showed that part of the dimer band observed under SDS-PAGE attributes to the disulfide bond formed between two C38 residues during the denaturing step and indicated that the rest of the dimer band is due to specific dimerization of NT *in vivo*.

To gain insights into domains responsible for trimerization without ‘contamination' of di- and tetramerization we thus utilized the Flag-tagged ΔNT mutant to construct plasmids with deletion of C-terminal fragments. We found that while the mutant with T622X truncation from ΔNT (named ΔNT/T622X) still forms trimers, the mutant with the entire CT deleted from ΔNT (named ΔNT/ΔCT) has no trimer (Fig. 3D). Since no cysteine residue is present in the CT, the observed trimer band likely corresponds to the specific trimierzation of PKD2L1 *in vivo*. Because domain CC2 is absent in these mutants, and ΔCT and T622X are dead (Fig. 2B) and functional mutants^24^, respectively, our data indicated that the C-terminal fragment E566-T622 contains a novel domain that is critical for both PKD2L1 trimerization and channel function.

### Identification of a C-terminal domain critical for both PKD2L1 homotrimerization and channel function

**Figure 4:**
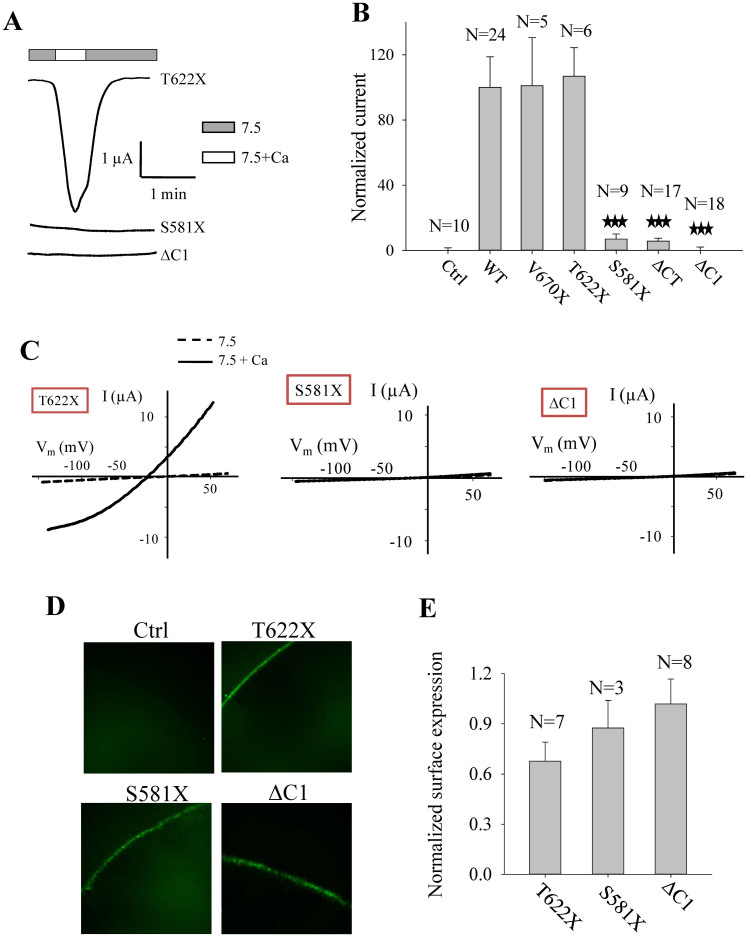
Identification of domain C1 of PKD2L1 critical for its channel function. (A) Representative whole-cell current tracings obtained from *Xenopus* oocytes expressing PKD2L1 truncation mutant T622X, S581X, or deletion mutant ΔC1 (C1 deletion from PKD2L1 WT) using the two-microelectrode voltage clamp technique under similar experimental conditions as those for Fig. 2. (B) Averaged currents obtained from oocytes expressing PKD2L1 WT or truncation/deletion mutants, as indicated. Currents at −50 mV were averaged from indicated numbers of oocytes and normalized to that of PKD2L1 WT. Water-injected oocytes were used as control (Ctrl). ‘***' indicates P ≤ 0.001 when comparing with “WT”. (C) Representative current–voltage relationship curves obtained using a voltage ramp protocol, as indicated in Fig. 2C, before (7.5) and after (7.5+Ca) addition of 5 mM CaCl_2_. (D) Representative immunofluorescence data showing the plasma membrane expression of mutants T622X, S581X and ΔC1 expressed in oocytes, or those injected with water (Ctrl). (E) Surface membrane expression of mutants T622X, S581X and ΔC1 were averaged from the indicated numbers of oocytes and normalized to that of PKD2L1 WT.

For WT or a mutant PKD2L1 channel to be functional it is necessary that they are in a correct oligomeric state. In an effort to identify a PKD2L1 domain that is essential for both trimerization and channel function we first made two deletion mutants with C1 or CC2 deleted from truncation mutant Flag-ΔNT (see Fig. 3A for a schematic illustration of the C1 and CC2 positions in CT), and performed WB experiments under the non-reducing condition. We found that while CC2 deletion significantly reduces the trimerization C1 deletion has a more substantial effect (Fig. 3E). Consistently, double deletion of C1 and CC2 completely abolished the trimer band. These data indicated that there exist two domains, C1 and CC2^22^,^23^ in CT, that are essential for trimerization. Human PKD2L1 C1 is a 48-amino-acid peptide containing 16 highly hydrophobic residues and 7 and 8 negatively and positively charged residues, respectively. Sequence alignment showed that C1 is the most conserved part in CT and shares overall 62.5% identity among different species (Fig. 3F), suggesting its importance in the PKD2L1 assembly and/or function. We next performed electrophysiology experiments using various C-terminal deletion and truncation mutations of PKD2L1. We found that any domain after T622 is not important for the channel function, as supported by functional mutant V670X and T622X channels. In contrast, truncation mutant S581X and the mutant with C1 deletion did not exhibit channel function (Fig. 4A–C). Our immunofluorescence data indicated that all these mutants target to the plasma membrane (Fig. 4D and E). These data together demonstrated that C1, but not CC2, is critical for PKD2L1 channel function. Thus, we have discovered for the first time a domain in PKD2L1, C1, that is essential for both homotrimerization and channel function. Interestingly, Ishimaru et al also found that PKD1L3/PKD2L1 M1-F621 responds to citric acid while PKD1L3/PKD2L1 M1-I560 shows no response, suggesting that domain C1 is also important for PKD1L3/PKD2L1 complex off-response channel function^25^.

### Further characterization of C1-involved trimerization

**Figure 5:**
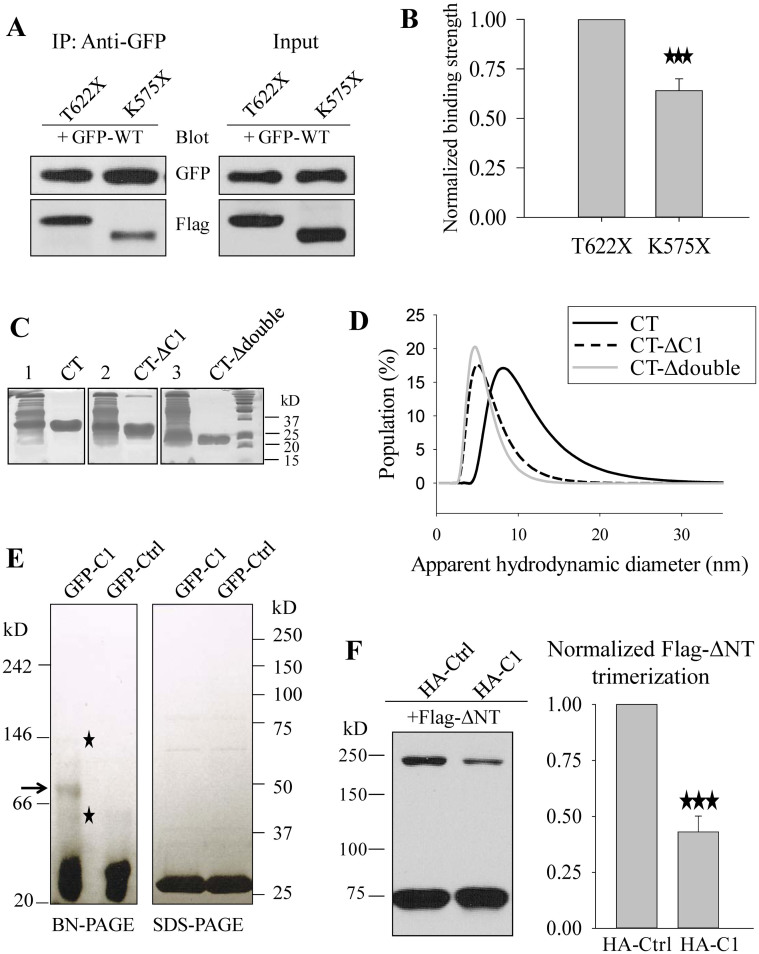
Further evidences for C1 involvement in trimerization. (A) Effect of C1 on the interaction between two PKD2L1 proteins assessed by co-IP. Left panel: representative data showing the interaction of GFP-PKD2L1 (GFP-WT) with Flag-T622X or Flag-K575X in HeLa cells. GFP-WT was first transfected, followed by transfection of T622X and K575X 12 hr later. Right panel: representative input data by WB showing the expression of WT, T622X and K575X. (B) Data from experiments in panel A were quantified, averaged, normalized, and then compared by paired t-test (***P ≤ 0.001, N = 3). (C) Coomassie blue staining of purified PKD2L1 C-terminus (CT, E566-S805), CT-ΔC1 (C1 deletion from CT), and CT-Δdouble (C1 and CC2 double deletion from CT) from *E. coli*. Data using crude lysates are shown on their left (lanes 1, 2 and 3). (D) Analysis of size distribution of purified CT, CT-ΔC1, and CT-Δdouble fragments by dynamic light scattering experiments. (E) Coomassie blue staining analysis of BN-PAGE or SDS-PAGE of purified protein GFP-C1 or GFP-Ctrl (human 4EBP1 M1-T50 fragment). Putative GFP-C1 trimer is indicated by an arrow on BN-PAGE. The faint bands below and above the putative trimer are indicated with stars. (F) Left panel: WB detection of over-expressed Flag-ΔNT with co-expression of HA-tagged blocking peptide C1 (HA-C1) or control peptide T622-E675 (HA-Ctrl) in HeLa cells under the non-reducing condition. 200 ng Flag-ΔNT plasmid and 1000 ng HA-C1 or HA-Ctrl plasmid were used in the co-transfection. Right panel: trimer bands were quantified, averaged, normalized, and compared by paired t-test (***P ≤ 0.001, N = 3).

To provide further documentations on the involvement of C1 in trimerization we first employed co-immunoprecipitation (co-IP) assays to determine the effect of C1 on the interaction between two differently tagged WT or mutant PKD2L1 proteins. To enhance the co-expression efficiency, we transfected HeLa cells with GFP-WT first and 12 hr later with Flag-T622X or -K575X. We collected cell lysates 30 hr after the second transfection. We found that the strength of the interaction of WT with K575X (that lacks C1), assessed by the immunoblotting band after normalization by input bands, is 36.5 ± 6.8% (N = 3, P < 0.001, by paired t-test) less than the interaction with T622X (Fig. 5A and B). We believe that the interaction between WT PKD2L1 and K575X is largely mediated through their N-terminus. In summary, our *in vitro* data were in support of the involvement of C1 in trimerization.

Further, we employed dynamic light scattering (DLS) to examine the role of C1 in the trimerization of purified CT in solution. DLS assesses a protein's apparent hydrodynamic diameter (D_H_) as being the size of a hypothetical hard sphere that diffuses in the same fashion as the hydrated protein being measured. We expressed and purified CT and its deletion mutants CT-ΔC1 (with C1 deletion) and CT-Δdouble (with both C1 and CC2 deletions) from *E. coli* (Fig. 5C). We found that the D_H_ of CT measured by DLS is 8.88 ± 0.26 nm (N = 6), which is significantly reduced to 5.80 ± 0.17 nm (N = 5, P < 0.001) and 5.49 ± 0.05 nm (N = 5, P < 0.001) for C1 and double deletions, respectively (Fig. 5D). Using the Mark-Houwink-Sakurada equation, molecular weight = a(D_H_)^b^, where parameters a and b were found to be 0.41 ± 0.05 and 2.48 ± 0.04, respectively, for proteins in the range of 17–440 kD^32^, the corresponding molecular weights deduced from these D_H_ values are 93 ± 6 kD, 32 ± 2 kD and 28 ± 1 kD, for CT, CT-ΔC1 and CT-Δdouble, respectively, indicating a predominant trimeric structure for CT and monomeric structures for the two deletion mutants under this experimental condition. These data indicated that C1 deletion significantly breaks down trimerization of CT, in support of the importance of C1 for oligomerization.

Next, we examined whether peptide C1 is sufficient to form trimers by itself. For this we first expressed C1 in and purified it from *E. coli*. Because C1 was not detectable when expressed alone, presumably due to its small size and/or instability, we utilized fusion protein GFP-C1 for *E. coli* expression. As a negative control, we expressed and purified GFP protein fused with human eukaryotic initiation factor 4E binding protein 1 (4EBP1) fragment M1-T50 (GFP-Ctrl) of a similar size. We then carried out BN-PAGE experiments which indeed revealed the presence of a trimer band for GFP-C1 but not for GFP-Ctrl (Fig. 5E). Of note, the trimer band was relatively weak, which may be due to the added coomassie blue G-250 that gave negative changes to C1 and reduced its trimerization during sample preparation for BN-PAGE. As a comparison and control, we carried out SDS-PAGE analysis as well and found no trimer band for C1 under this condition (Fig. 5E). Therefore, our data showed that purified C1 itself forms trimers. We also noted visible weak bands below and above the trimer band, which may represent a dimer due to the well-known weak dimerization of GFP and a hexamer due to GFP dimerization plus C1 trimerization, respectively.

### Role of C1 as a blocking peptide

**Figure 6:**
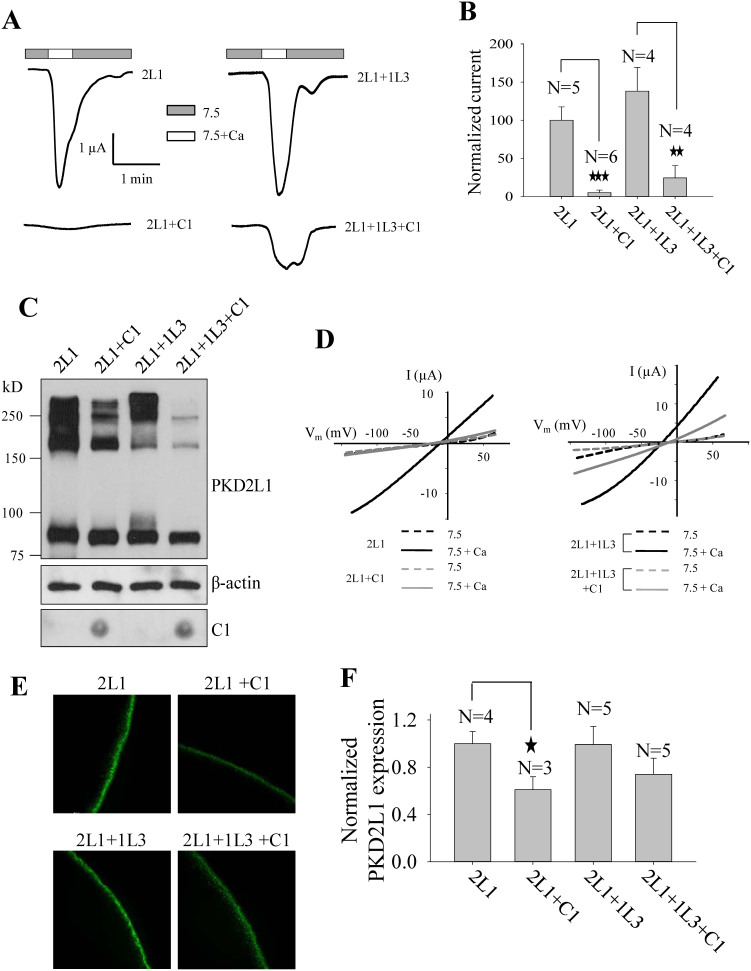
Effect of C1 on PKD2L1 trimerization and function. (A) Representative whole-cell current tracings from *Xenopus* oocytes expressing PKD2L1 (2L1) or PKD2L1/PKD1L3 (2L1+1L3), with or without C1 using the two-microelectrode voltage clamp technique under similar conditions as for Fig. 2. (B) Averaged currents elicited by extracellular 5 mM Ca^2+^ from oocytes expressing 2L1 or 2L1+1L3, with or without C1, and voltage clamped at −50 mV. Currents were averaged from different numbers of oocytes, as indicated, and normalized to that of WT PKD2L1 alone. (C) WB detection of PKD2L1 expression or dot blot detection of C1 expression in oocytes. (D) Representative current–voltage relationship curves obtained using a voltage ramp protocol, as indicated in Fig. 2C, before (7.5) and after (7.5+Ca) addition of 5 mM CaCl_2_. (E) Representative immunofluorescence data showing the oocyte plasma membrane expression of 2L1 and 2L1+1L3, with or without C1. (F) Surface expression was quantified and averaged from the indicated numbers of oocytes, and normalized to that of 2L1.

We reasoned that C1 may disrupt the oligomerization of full-length PKD2L1 through competitive binding, thereby inhibiting channel function. To test this, we applied a similar blocking peptide strategy that we described previously^9^. We first co-transfected PKD2L1 ΔNT with HA-tagged C1 or control plasmid encoding HA-T622-E657 (negative Ctrl) that has no overlap with C1 or CC2. Indeed, we found that co-expression of C1 decreases ΔNT trimerization to 42.7 ± 7.5% (N = 3, P < 0.001, by paired t-test) (Fig. 5F). Next, using oocyte expression and electrophysiology, we found that expression of C1 substantially inhibits the channel function of PKD2L1 alone and complex PKD2L1/PKD1L3 (Fig. 6A, B and D). Our WB assays using similarly prepared oocytes showed that C1 expression significantly reduces the formation of PKD2L1 oligomers but not monomers, regardless whether PKD1L3 was co-expressed or not (Fig. 6C). Of note, due to its small size of only about 5 kD, we carried out dot blot assays and confirmed its expression (Fig. 6C). Our immunofluorescence data indicated that over-expression of C1 reduces the PKD2L1 surface expression (Fig. 6E and F), which may be the reason why reduction in the trimer band did not result in an increase in the monomer band in our WB experiments (Fig. 6C).

## Discussion

Because the function of an ion channel presumably relies on its intact oligomeric state deletion of or alterations in an oligomerization domain would substantially affect the channel architecture and consequently channel activity. Up to now it has remained unknown as to which domains of PKD2L1 that are important for both oligomerization and function. Previous studies identified CC2 as a trimeric domain in CT that is important for the surface membrane expression of PKD2L1 and complex PKD2L1/PKD1L3^21^,^22^,^23^. However, using the *Xenopus* oocyte expression system, it was previously shown that PKD2L1 with truncation either before or after the CC2 domain does not significantly affect the channel function^24^, which is also confirmed by the current study (Fig. 4B), indicating that CC2 is not required for PKD2L1 channel function. The reported 3:1 oligomeric assembly for PKD2L1/PKD1L3 channel complex and our current *in vivo* data from mouse tissues showing the presence of trimeric assembly indicate the presence of a yet to-be-identified domain(s) important for a functional PKD2L1 trimeric state. Indeed, our current studies using a combination of *in vitro* expression in mammalian cells and *Xenopus* oocytes, electrophysiology, mutagenesis, protein-protein interaction, and dynamic light scattering identified the C-terminal C1 domain that is the most conserved in CT and critical for both PKD2L1 trimerization and channel function.

Our non-reducing SDS-PAGE with mouse tissues showed the same oligomers pattern in kidney, testis and brain and these oligomers were reduced to monomer with reducing agents (Fig. 1A). This would suggest that all PKD2L1 oligomers in the tissues are generated through cysteine-cysteine disulfide bonds since reducing agents are expected to only disrupt oligomerization mediated by disulfide bonds. However, for some unknown reasons, reducing agents may also disrupt oligomerization mediated by peptide-peptide interactions, at least for some multiple-transmembrane proteins. For example, Feng et al showed that dimers of PKD2 truncation mutant PKD2-L703X detected with non-reducing SDS-PAGE disappeared by the addition of reducing agents and that these dimers are indeed formed by a peptide-peptide interaction in the N-terminus of PKD2^26^. Further, oligomers formed through peptide-peptide interactions under non-reducing SDS-PAGE may be more vulnerably disrupted by reducing agents in tissue lysates than in cell line lysates for unclear reasons. For example, almost all PKD2 oligomers were reduced into monomers with addition of reducing agents in human kidney tissues, but the effect of reducing agents were only moderate in cell lines^33^. In the case of PKD2L1, previous studies by other groups have reported the presence of PKD2L1 homooligomers through its C-terminal peptide-peptide interactions, eg, through CC2 trimerization^21^ and PKD2L1/PKD1L3 heterooligomerization at 3:1 stoichiometry through peptide-peptide interaction^25^. Together with our current data on its C-terminal C1 trimerization, we think that part of PKD2L1 oligomers detected with non-reducing SDS-PAGE using tissue lysates should be mediated by peptide-peptide interactions, possibly the C1-C1 interaction, and reduced to monomers by reducing agents.

TRPC, TRPM and TRPN channels were found to contain a conserved intracellular TRP domain downstream of TM6, which is similarly located as C1. This TRP domain was proposed to participate in subunit assembly and/or allosteric modulation of channel gating^34^. Interestingly, the recently resolved TRPV1 structure showed that charged side chains within the TRP domain interact with pre-S1 helix in the N-terminus through hydrogen bonding and salt bridging^35^. It is possible that C1 shares some functional similarities and plays corresponding roles in PKD2L1. Eg, in addition to its trimerization role, it seemed to regulate PKD2L1 dimerization formed by PKD2L1 NT (see Fig. 2F). Future studies will need to address this by providing more direct evidence.

We wondered why PKD2L1 needs two distinct trimerization domains in CT of PKD2L1. PKD2L1 channel is activated by Ca^2+^ followed by channel inactivation, presumably through an increase in the intracellular Ca^2+^ concentration^8^, but it remains unclear whether Ca^2+^ regulates PKD2L1 channel activity through direct binding. CT contains a Ca^2+^-binding EF hand motif (E637-L665) that negatively regulates the ability of PKD2L1 channel to be activated by Ca^2+^, suggesting that Ca^2+^ binds to this domain to prevent the channel from over-activation^24^. Direct binding of Ca^2+^ to CT was indeed reported^23^ although it remains unknown whether and how this binding is relevant to channel function. At least, this same study showed that Ca^2+^ binding is not affected by mutations of 6 residues to alanine within CC2, suggesting that CC2 is not involved in Ca^2+^ binding. More importantly, our previous and current studies both demonstrated that CC2 is not important for PKD2L1 channel function. Although arguably, CC2 was found to be important for surface expression of PKD2L1 and complex PKD2L1/PKD1L3 in *Xenopus* oocytes^21^ whether it promotes the trafficking to, or prevents the retrieval from, the surface membrane remains unknown. In contrast, although C1 did not seem to affect the PKD2L1 surface membrane expression (Fig. 4E) it is critical for the channel function. It is possible that nature chooses to have two trimerization domains that together ensure proper surface membrane configuration and stability, as well as channel function.

**Figure 7:**
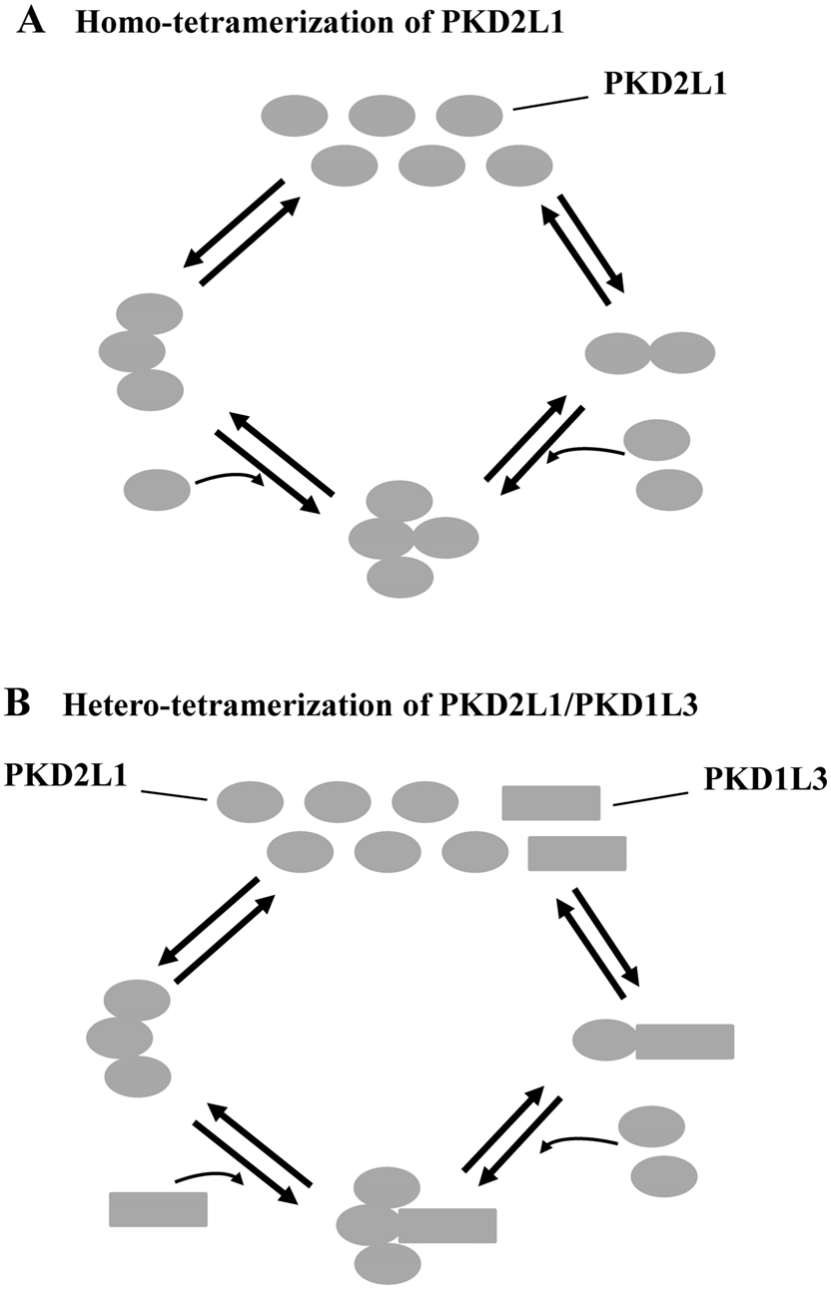
Model illustrating how a PKD2L1 homo- or heterotetramerer can be formed. (A) A PKD2L1 (oval) homotetramer can be formed either through first homotrimerization followed by recruitment of a fourth subunit to the trimer by dimerization or through first homodimerization followed by recruitment of two subunits to form a trimer with a subunit in the dimer. Of notes, 1) the illustration is only to indicate trimeric and dimeric binding and does not intend to show how the pore region is organized; and 2) the drawn trimer does not intend to mean that there are two points of contact. (B) A PKD2L1 heterotetramer can be formed with a PKD1 homologue (square), eg, PKD1L3 or PKD1L1, through first trimerization of PKD2L1 and then recruitment of a PKD1 homologue, or reversely, through first heterodimerization between PKD2L1 and a PKD1 homologue, followed by recruitment of two PKD2L1 subunits to form a trimer with the existing PKD2L1 subunit.

Members of the TRP superfamily including PKD2 and PKD2L1 are assumed to assemble into homo- and/or hetero-tetramers with either a PKD1 homologue or another TRP protein, but the molecular mechanisms underlying these assemblies are still unclear. Like PKD2L1, the PKD2 C-terminus was found to form trimers^20^,^22^. The trimerization of the PKD2 or PKD2L1 C-termini seems to nicely explain the tetrameric organization of the PKD2/PKD1 or PKD2L1/PKD1L3 complex with a trimer/monomer assembly^20^,^21^. Based on our current data we propose that a functional PKD2L1 homotetramer can be formed either through C-terminal trimerization followed by N-terminal dimerization of a fourth subunit with a subunit in the trimer or through dimerization followed by trimerization (see model in Fig. 7). Thus, we think that, under physiological conditions, depending on the availability of a co-localized PKD1 homologue, PKD2L1 forms homo- or heterotetramers by a shared way, namely, homotrimerization of PKD2L1 and heterodimerization of PKD2L1-PKD1 homologue (Fig. 7). We should however bear in mind that interaction forces involved in the organization of TMs for a pore forming architecture are important as well although they are not discussed here.

It is noted that such a 3 + 1 assembly protocol for forming homo- and heterotetramers has been reported for other channels. Eg, the cytoplasmic C-terminal leucine zipper domain (G488-Y526) of small conductance voltage-gated Ca^2+^-activated K^+^ channel (SK_Ca_) forms trimers, as shown by crystallographic analyses^36^ while the functional full-length SK_Ca_ channel forms homotetramers^37^. Similarly, a C-terminal domain (R583-D611) of voltage-gated K^+^ channel K_v_7.1 was shown to trimerize but the full-length channel forms tetramers^38^. Under physiological conditions, cyclic-nucleotide-gated channels are more likely heterotetramers consisting of the A and B subtypes with the A-trimer:B-monomer assembly when both are present. In cells over-expressing the A subtype, homotetramers were found^39^,^40^,^41^. Therefore, PKD2L1 as a TRP superfamily member may possess a strategy of oligomeric assembly that is shared by other families of ion channel.

In summary, our study has found that endogenous PKD2L1 in mouse tissues exhibits oligomeric states including dimers, trimers and tetramers. We identified a novel PKD2L1 C-terminal C1 domain that is associated with stronger trimerization than the previously reported CC2 domain. Importantly, C1 represents the first oligomerization domain critical for PKD2L1 channel function. We thus propose that functional PKD2L1 channel assembly involves both trimerization and dimerization, a mechanism possibly shared by some non-TRP types of ion channel.

## Methods

### Plasmids construction

Human full-length PKD2L1 cDNA was amplified from pCHGF^9^ by PCR and subcloned into pcDNA3.1(+) and pEGFP-C2 for mammalian cell expression. Flag tag was inserted 5' of the PKD2L1 coding region in pcDNA3.1 (+). Mouse PKD1L3 plasmid was a kind gift of Dr. H. Matsunami from Duke University. All mutations were made with QuikChange Lightning Site-Directed Mutagenesis kit (Agilent Technologies, La Jolla, CA) and confirmed by sequencing.

### Cell culture and transfection

HeLa and HEK cells were cultured in Dulbecco's modified Eagle's medium supplemented with 10% fetal bovine serum (FBS) and 1% penicillin/streptomycin (Sigma, St. Louis, MO). Cells of less than 25 passages were cultured to full confluence before collection. Transient transfection was performed on cells cultured to 70%–90% confluence using Lipofectamine 2000 (Invitrogen, Carlsbad, CA) according to the manufacturer's instruction.

### WB

Protein samples were separated on 8% SDS-PAGE gels. For the non-reducing SDS-PAGE, samples were mixed with SDS loading buffer without reducing reagents and kept on ice for 5 minutes (min) prior to electrophoresis. For the reducing SDS-PAGE, samples were mixed with loading buffer supplemented with 0.5 M DTT or 5% 2-mercaptoethanol (2-ME), and subject to heating at 65°C for 5 min. 30 μg of total protein was loaded per lane. Flag and GFP (B-2) antibodies were purchased from Santa Cruz Biotechnology (Santa Cruz, CA). PKD2L1 antibody (H00009033) was purchased from Abnova (Taipei, Taiwan) for tissue detection. Rabbit antibody against PKD2L1 (PR71) was custom made and used previously^42^. Secondary antibodies were purchased from GE Healthcare (Baie d'Urfe, QC, Canada). Band intensity was analyzed with the software ImageJ (NIH, Bethesda, MD).

### BN-PAGE

HeLa cell lysates for BN-PAGE were prepared with NativePAGE Sample Prep Kit (Invitrogen) according to the manufacturer's protocol. Coomassie blue G-250 was added to supernatants at 8:1 detergent:G-250 ratio. Protein complexes were separated at 150 V for 90 min using NativePAGE Novex 3–12% BisTris gels (Invitrogen). For immunoblotting, gel was incubated in 20 mM Tris-HCl (pH 8.3), 0.15 M glycine, and 0.02% SDS for 5 min at room temperature. Proteins were then transferred to polyvinylidene difluoride membranes (at 150 mA for 90 min and 4°C). Membranes were blocked with 3% skimmed milk in PBS buffer with 0.1% tween-20 for 40 min at room temperature and then incubated with antibodies.

### Dot blot

Lysates of *Xenopus* oocytes were spotted onto a nitrocellulose membrane and then let the membrane dry. Glutaraldehyde fixation was applied as previously described^43^ to enhance the retention of short peptides on the membrane. Briefly, membrane was immersed in PBS containing 0.5% (v/v) glutaraldehyde for 5 min and moved to a fresh PBS/glutaraldehyde solution for 10 min, followed by placement in PBS containing 50 mM glycine to stop cross-linking reaction. Membrane was then washed once with PBS buffer and subjected to normal WB procedure.

### Immunofluorescence

*Xenopus* oocytes were washed in PBS, fixed in 3% paraformaldehyde for 15 min, washed 3 times in PBS plus 50 mM NH_4_Cl, and then permeabilized with 0.1% Triton X-100 for 4 min. Oocytes were then washed 3 times in PBS for 5 min each time, blocked in 3% skim milk in PBS for 30 min, and then incubated overnight with the rabbit anti-PKD2L1 polyclonal antibody (cat# PAB5914, Abnova). This was followed by 3 times 10-min washes in PBS. Oocytes were then incubated with a secondary AlexaFluor 488-conjugated donkey anti-rabbit antibody (Jackson ImmunoResearch Laboratories, West Grove, PA) for 30 min, followed by 3-time washes in PBS and mounting in Vectashield (Vector Labs, Burlington, ON). The slides were examined on an AIVI spinning disc confocal microscopy (Cell Imaging Facility, Faculty of Medicine and Dentistry, University of Alberta). Plasma membrane intensity of WT or mutant PKD2L1 was assessed by quantifying the plasma membrane immunofluorescence using Volocity 6.2 (Perkin Elmer, Waltham, MA). Background fluorescence was subtracted and data were normalized to the average PKD2L1 WT intensity.

### Co-IP

Co-IP was performed using lysates of HeLa cells over-expressing GFP-PKD2L1 and Flag-tagged mutant PKD2L1 constructs. HeLa cell monolayer in 100-mm dishes was washed twice with PBS and solubilized in ice-cold CellLytic-M lysis buffer supplemented with proteinase inhibitor mixture (Sigma). Supernatants were collected following centrifugation at 16,000 × *g* for 15 min. Equal amounts of total proteins from postnuclear supernatants were pre-cleared for 1 hour (hr) with protein G-Sepharose (GE Healthcare), and then incubated for 4 hr at 4°C with the antibody against GFP. After the addition of 100 μl of 50% protein G-Sepharose, the mixture was incubated overnight with gentle shaking at 4°C. The immune complexes absorbed to protein G-Sepharose were washed five times with Nonidet P-40 lysis buffer (50 mM Tris, pH 7.5, 150 mM NaCl, 1% Nonidet P-40) with proteinase inhibitor and eluted by SDS loading buffer. Precipitated proteins were analyzed by WB using the antibodies against Flag or GFP.

### Protein expression in and purification from *E. coli*

DNA fragment encoding C1 was inserted into pET28a(+) that contains upstream GFP gene. BL21 (DE3) strain (Novagen, Darmstadt, Germany) was used for expression. Following growth at 37°C to an optical density (OD) of 0.6, cultures were cooled to 30°C, induced with 1 mM isopropyl β-D-thiogalactoside and incubated for 6 hr. Cell lysates from 50 mL culture were prepared with CellLytic-B lysis buffer (Sigma) according to the manufacturer's instruction. Proteins were pulled down with Ni-NTA resin (Qiagen, Venlo, Netherlands) and eluted from beads with 250 mM imidazole by following the manufacturer's manual.

### Preparation of mRNAs and microinjection into oocytes

Capped mRNAs of WT or mutant PKD2L1 were synthesized by *in vitro* transcription from a linearized template in the pCHGF vector using the mMESSAGE mMACHINE1 kit (Ambion, Austin, TX). Stage V–VI oocytes were isolated from *Xenopus laevis*. Defolliculation of oocytes was performed through incubation in Ca^2+^-free Barth's solution^24^ containing collagenase (2 mg/ml) at RT for 2–2.5 hr. Oocytes were then incubated at 18°C in the Barth's solution for at least 3 hr before injection of 50 nl RNase-free water containing 50 ng mRNAs. An equal volume of water was injected into each control oocyte. The present study was approved by the Ethical Committee for Animal Experiments of the University of Alberta, and was carried out in accordance with the Guidelines for Research with Experimental Animals of the University of Alberta and the Guide for the Care and Use of Laboratory Animals (NIH Guide) revised in 1996. Injected oocytes were incubated at 16–18°C in the Barth's solution supplemented with antibiotics for 2–4 days prior to experiments.

### Two-microelectrode voltage clamp

Two-microelectrode voltage clamp experiments were performed as described before^9^. Briefly, the two electrodes (Capillary pipettes, Warner Instruments, Hamden, CT) impaling oocytes were filled with 3 M KCl to form a tip resistance of 0.3–2 MΩ. The standard extracellular solution (pH 7.5) containing 100 mM NaCl, 2 mM KCl, 1 mM MgCl_2_ and 10 mM HEPES was used. The solution containing extracellular Ca^2+^ was prepared from the standard solution with the addition of CaCl_2_ to a final concentration of 5 mM. Duration of application of Ca^2+^ medium was indicated in time course recordings. Oocyte whole-cell currents were recorded using a Geneclamp 500B amplifier and Digidata 1322A AD/DA converter (Molecular Devices, Union City, CA). The pClamp 9 software (Axon Instruments, Union City, CA) was applied for data acquisition and analysis. Currents and voltages were digitally recorded at 200 μs/sample and filtered at 2 kHz through a Bessel filter. SigmaPlot 12 (Systat Software, San Jose, CA) was used for data fitting and plotting.

### DLS

DLS experiments were performed using a Malvern Zetasizer Nano ZS instrument (Malvern, Worcestershire, UK) at 25°C, as described previously^44^. Freshly purified PKD2L1 CT and deletion mutants from *E. coli* in a solution containing 50 mM NaH_2_PO4, 300 mM NaCl and 250 mM imidazole, pH 8.0 were passed through a 0.22 μm filter to remove large particles or aggregates and diluted to 0.4 mg/ml before measurements. Correlation data obtained with DynaLS software (Malvern) were fitted using SigmaPlot 12 (Systat Software) to derive the average apparent hydrodynamic diameter.

### Statistic analysis

Data were analyzed and plotted using SigmaPlot 12 (Systat Software), and expressed as mean ± SEM (N), where SEM stands for the standard error of the mean and N indicates the number of experimental repeats. Paired or unpaired Student t-test was used to compare two sets of data. A probability value (P) of less than 0.05, 0.01 and 0.001 was considered statistically significant and indicated by “*”, “**” and “***”, respectively.

## Author Contributions

Conceived and designed the experiment: W.Z., C.F.P., Y.C., H.B.Z., J.F.T. and X.Z.C. Performed the experiments: W.Z., S.H., J.W.Y., J.H., F.Z. and S.H.A. Analyzed the data: W.Z., C.F.P., Y.C., H.B.Z., J.F.T. and X.Z.C. Wrote the paper: W.Z. and X.Z.C.
